# Severe Hyponatremia Secondary to Pituitary Macroadenoma With Internal Hemorrhage in the Postpartum Period: A Case Report

**DOI:** 10.7759/cureus.66414

**Published:** 2024-08-07

**Authors:** David Ahn, Hema Narlapati, Robert D Leimbach

**Affiliations:** 1 Department of Internal Medicine, Tripler Army Medical Center, Honolulu, USA; 2 Department of Endocrinology, Tripler Army Medical Center, Honolulu, USA

**Keywords:** pituitary apoplexy, multidisciplinary care, visual disturbances, postpartum period, profound hyponatremia, pituitary macroadenoma

## Abstract

Pituitary macroadenomas, especially those complicated by internal hemorrhage (pituitary apoplexy), can lead to severe endocrine dysfunction and visual disturbances. This is particularly challenging to diagnose in the postpartum period due to physiological changes associated with childbirth and breastfeeding. This case report aims to highlight the diagnostic and therapeutic complexities of managing severe hyponatremia and visual changes in a woman with a pituitary macroadenoma in the postpartum period.A 34-year-old female, five months postpartum, presented with a one-month history of intermittent nausea, headaches, and blurry vision, which worsened over the past week. Initial laboratory results revealed severe hyponatremia with a sodium level of 112 mEq/L. Imaging studies, including MRI, confirmed a 1.9 x 1.8 x 1.7 cm pituitary macroadenoma with internal hemorrhage exerting mass effect on the optic chiasm. The patient was managed with hypertonic saline for hyponatremia and empiric glucocorticoid supplementation for suspected adrenal insufficiency. A multidisciplinary approach involving endocrinology, neurosurgery, and ophthalmology was employed to address her complex medical needs. This case underscores the importance of considering pituitary pathology in women presenting with severe hyponatremia and visual changes postpartum. A multidisciplinary approach is essential for optimal management and prevention of long-term complications. Early recognition and appropriate intervention are crucial in ensuring a favorable outcome.

## Introduction

Pituitary macroadenomas are significant clinical entities that can lead to severe endocrine dysfunction and visual disturbances, especially when complicated by internal hemorrhage, known as pituitary apoplexy. These adenomas are typically benign tumors greater than 10 mm in size and can cause symptoms due to hormone overproduction or compression of surrounding structures. Common symptoms of pituitary adenomas include headaches, visual disturbances (such as bitemporal hemianopsia due to optic chiasm compression), hormonal imbalances (such as hyperprolactinemia, Cushing's disease, or acromegaly), fatigue, and unexplained weight gain or loss. Presentation may vary depending on the type of hormone secreted by the tumor or the degree of mass effect exerted on adjacent tissues [1,2].

During pregnancy, a series of changes that impact the pituitary gland occur. Physiological hormonal secretion from the placenta mediates an increase in the ovarian production of estrogen and progesterone. The surge in estrogen levels stimulates the pituitary gland and leads to the hypertrophy and hyperplasia of lactotroph cells [3].

Pituitary apoplexy is a medical emergency characterized by sudden hemorrhage or infarction of the pituitary gland, often within a pre-existing adenoma. This condition can lead to acute deterioration in vision, severe headache, ophthalmoplegia, altered mental status, and hormonal deficiencies, driven by rapid tumor expansion from internal bleeding, compressing the pituitary gland and its blood vessels. In the postpartum period, this risk is heightened by several factors. Pregnancy increases blood flow to the pituitary gland to support elevated hormonal activity, leading to vascular fragility. The gland enlarges due to increased hormone production, such as prolactin, making it more prone to hemorrhage, particularly if an adenoma is present. Childbirth, being a significant physical stressor, involves rapid hemodynamic changes that can trigger apoplexy. The physiological stress and trauma of childbirth can elevate intracranial pressure, causing bleeding in a susceptible pituitary gland [4].

Women in the postpartum period present unique diagnostic challenges due to physiological changes associated with childbirth and breastfeeding. This case report illustrates the importance of considering pituitary pathology in patients presenting with severe postpartum hyponatremia and visual changes. The purpose of this report is to highlight the diagnostic and therapeutic complexities involved and to underscore the importance of a multidisciplinary approach in managing such cases.

## Case presentation

A 34-year-old female, G3P3, presented with a one-month history of intermittent nausea, headaches, and blurry vision. These symptoms had become more persistent over the past week, accompanied by a single episode of vomiting. She described the blurry vision as a shift from near to farsightedness at approximately six inches from her face, with no symptoms indicative of bitemporal hemianopsia. The patient had recently completed a course of oral Augmentin for a stye. She had a history of two cesarean sections, gestational thrombocytopenia, and a postpartum hemorrhage (PPH) during her last cesarean delivery. She was breastfeeding her five-month-old child and had been advised to increase her water intake to enhance milk production.

The patient presented to the Emergency Department (ED) due to her vision changes, prompted by her husband. Initial blood pressure was 121/85 mmHg. Initial laboratory results revealed severe hyponatremia with a sodium (Na) level of 112 mEq/L. Other laboratory findings included hypochloremic non-anion gap metabolic acidosis (NAGMA), leukopenia with neutropenia and lymphopenia, and thrombocytopenia. Her spot urine sodium was 115 mmol/L, urine osmolality was 427 mOsm/Kg, and serum osmolality was 229.0 mOsm/kg. A non-contrast head CT (NCHCT) revealed a 1.7 x 1.6 cm pituitary macroadenoma with suspected compression of the optic chiasm. The patient received an initial 100 cc bolus of 3% hypertonic saline. The subsequent sodium level was 111 mEq/L. Further management included the continuous administration of 3% hypertonic saline at 30 cc/hour, with close monitoring of serum sodium levels every two hours. Free water restriction and concentrated juices were allowed to expedite the correction of hyponatremia.

**Figure 1:**
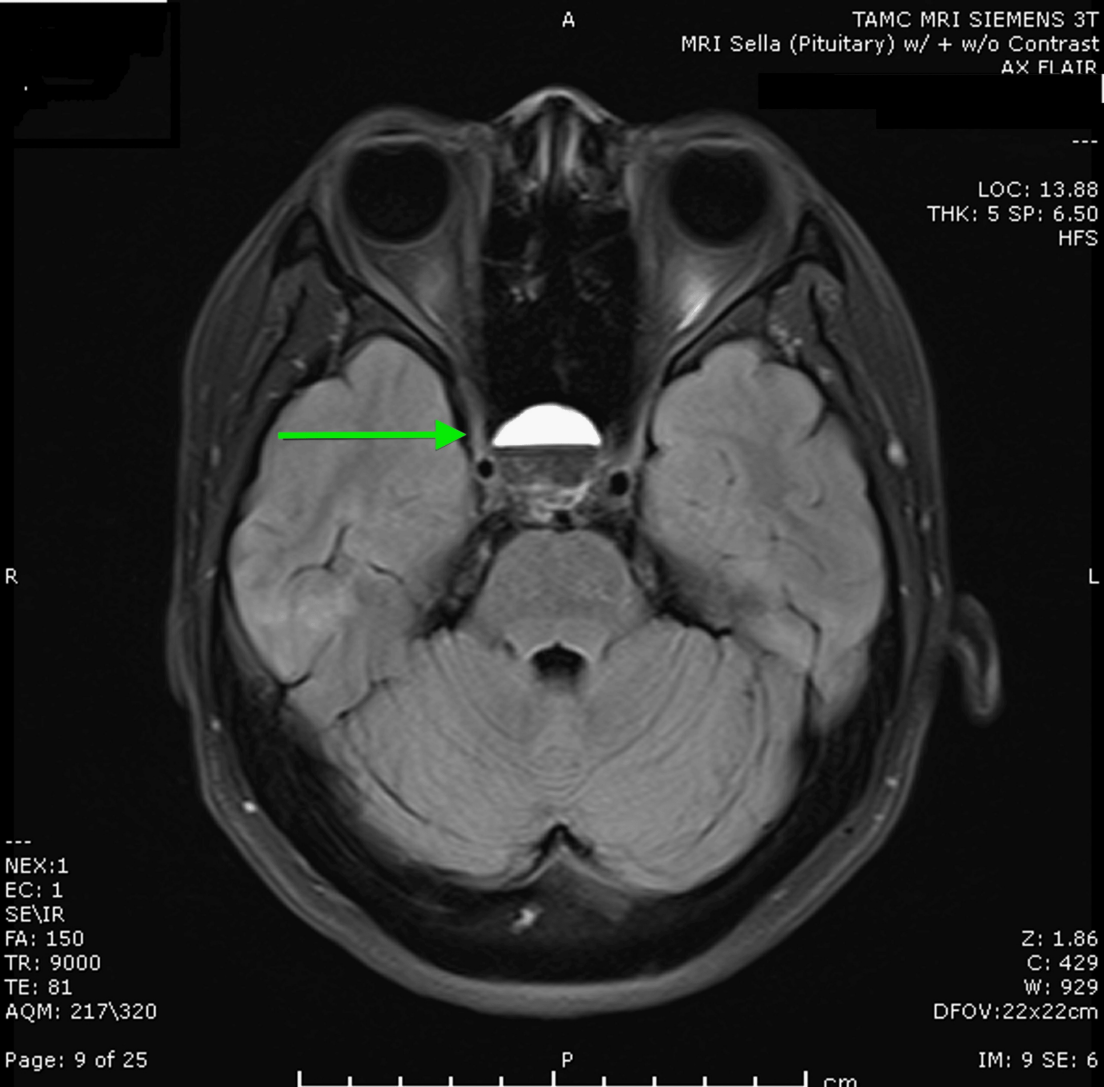
Axial FLAIR MRI image of the pituitary gland showing a hyperintense lesion within the pituitary macroadenoma, consistent with internal hemorrhage. FLAIR: fluid-attenuated inversion recovery

**Figure 2:**
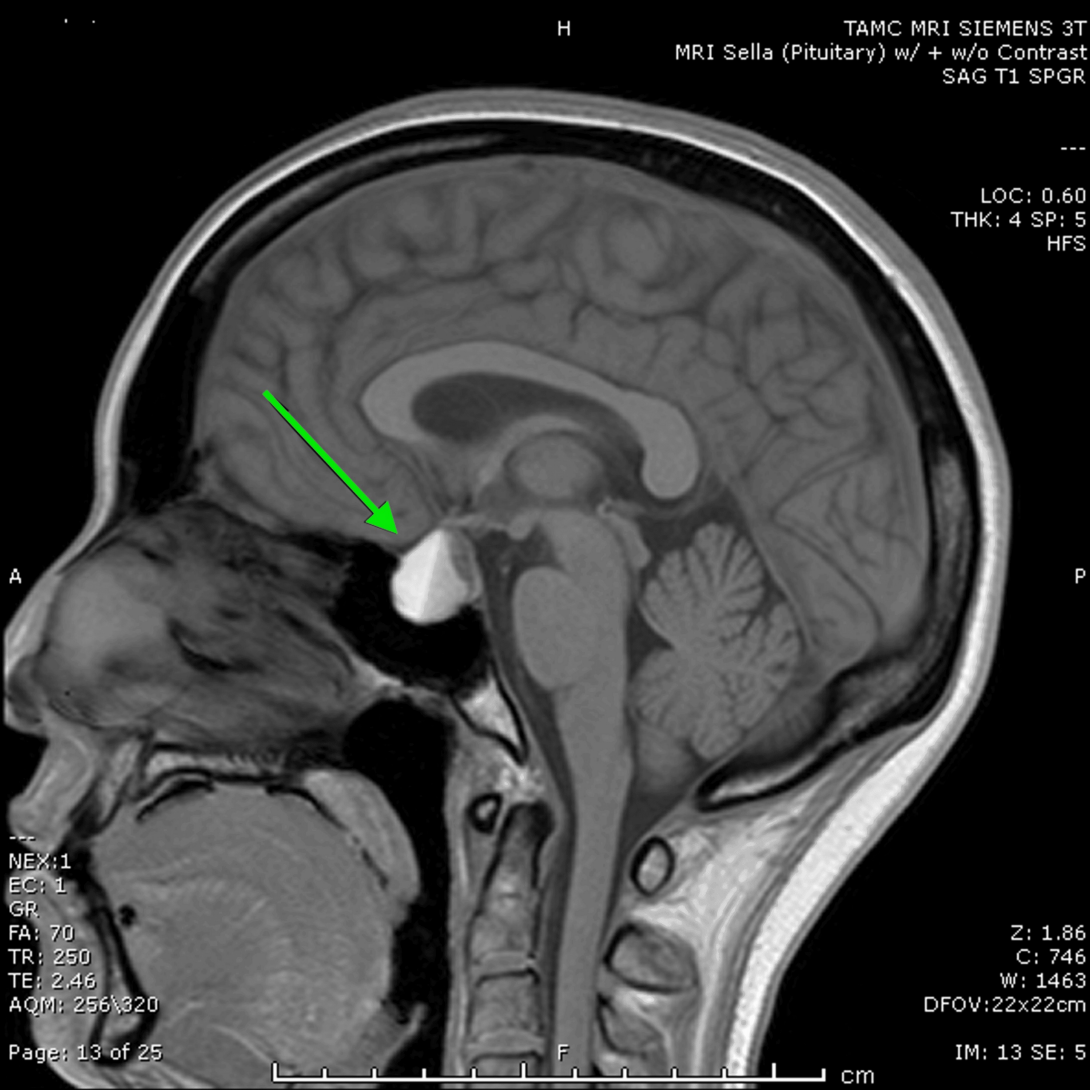
Sagittal T1-weighted MRI image demonstrating a large pituitary mass with internal hemorrhage, exerting mass effect on the optic chiasm. Note the demarcation indicating the fluid-fluid level, with the hyperintense region representing acute to subacute bleed and the hypointense region likely representing serous or proteinaceous fluid.

An MRI of the brain with and without contrast confirmed the presence of a 1.9 x 1.8 x 1.7 cm pituitary macroadenoma (Figures 1, 2) with internal hemorrhage. The mass demonstrated extension into the sphenoid and cavernous sinuses, exerting a mass effect on the optic chiasm (Figure 2). The imaging findings were consistent with pituitary apoplexy, characterized by the acute hemorrhage within the macroadenoma. An endocrinological assessment revealed a cortisol level of 12.5 mcg/dL. Given the profound hyponatremia and the presence of a macroadenoma, empiric glucocorticoid supplementation was initiated. The patient received hydrocortisone 100mg IV, followed by a planned tapering regimen of hydrocortisone 25 mg every six to eight hours. Additional pituitary hormone evaluations are shown in Table 1. She also had an undetectable estradiol of less than 10 pg/mL with follicle-stimulating hormone (FSH) of 2.49 mIU/mL and luteinizing hormone (LH) of 0.12 mIU/mL, likely due to the medroxyprogesterone injection she received one month ago for contraceptive purposes.

**Table 1:** Laboratory results ACTH: adrenocorticotropic hormone; DHEA-S: dehydroepiandrosterone sulfate; IGF1: insulin-like growth factor 1; TSH: thyroid stimulating hormone; FSH: follicle stimulating hormone; LH: luteinizing hormone

Test	Result	Normal Range
Sodium (Na)	112 mEq/L	135-145 mEq/L
Spot Urine Sodium	115 mmol/L	20-220 mmol/L
Urine Osmolality	427 mOsm/kg	300-900 mOsm/kg
Serum Osmolality	229.0 mOsm/kg	275-295 mOsm/kg
ACTH	14.4 pg/mL	7.2-63.3 pg/mL
DHEA sulfate	32.3 mcg/dL	84.8-378 mcg/dL
IGF1	148 ng/mL	84-281 ng/mL
Prolactin	76.8 ng/mL	2-30 ng/mL
TSH	0.3305 uIU/mL	0.35-5.0 uIU/mL
Total T3	0.46 ng/mL	0.5-1.6 ng/mL
Free T4	1.02 ng/dL	0.8-1.6 ng/dL
Estradiol	<10 pg/mL	30-400 pg/mL
FSH	2.49 mIU/mL	2.8-11.3 mIU/mL
LH	0.12 mIU/mL	1.1-11.6 mIU/mL
Serum Sodium (follow-up, 5 days)	138 mmol/L	135-145 mmol/L
Serum Osmolality (follow-up, 5 days)	285 mOsm/kg	275-295 mOsm/kg
TSH (follow-up, 5 days)	0.35 uIU/mL	0.35-5.0 uIU/mL
DHEA-S (follow-up, 2 weeks)	76.4 mcg/dL	84.8-378 mcg/dL
Cortisol (cosyntropin test, 1 month) - initial	10.3 mcg/dL	5-25 mcg/dL
Cortisol (cosyntropin test, 1 month) - stimulated	20.9 mcg/dL	>18-20 mcg/dL
Serum Sodium (follow-up, 1 month)	141 mmol/L	135-145 mmol/L

Neurosurgery was consulted, and it was determined that no immediate surgical intervention was necessary. An ophthalmology consultation was scheduled to formally evaluate the patient's visual field deficits. The patient’s leukopenia, neutropenia, lymphopenia, and thrombocytopenia were investigated for potential causes. A peripheral smear was obtained, and tests for vitamin B12, folate, and copper levels were conducted. The leukopenia and thrombocytopenia were suspected to be reactive, possibly related to the recent antibiotic use. 

The patient was discharged on hydrocortisone 15 mg daily (administered as 10 mg in the morning and 5 mg in the early afternoon). Follow-up five days after discharge revealed normal serum sodium of 138 mmol/L, normal serum osmolality, and normal thyroid axis. Repeat dehydroepiandrosterone sulfate (DHEA-S) two weeks after discharge was 76.4 mcg/dL. A cosyntropin stimulation test performed at the one-month follow-up showed an initial cortisol of 10.3 mcg/dL, which stimulated to 20.9 mcg/dL. Repeat serum sodium was 141 mmol/L.

## Discussion

Pituitary apoplexy is a rare but serious complication of pituitary macroadenomas, often presenting with acute headache, visual disturbances, and endocrine dysfunction [5]. In the postpartum period, these symptoms can be easily attributed to postpartum physiological changes, leading to potential delays in diagnosis [6,7]. This case illustrates the importance of considering pituitary pathology in women in the postpartum period presenting with severe hyponatremia and visual changes. The patient's profound hyponatremia was exacerbated by increased water intake for breastfeeding, compounded by syndrome of inappropriate antidiuretic hormone secretion (SIADH) induced by the macroadenoma [8]. Empiric glucocorticoid supplementation was crucial, given the high risk of adrenal insufficiency in patients with pituitary apoplexy [9].

The classic presentation of acute pituitary apoplexy, which includes sudden headache, visual deficit, hypopituitarism, and impaired consciousness, is relatively rare, occurring in less than 10% of patients with macroadenomas [1]. More common is subacute pituitary apoplexy, which can present with minimal or no symptoms and occurs in up to 25% of pituitary macroadenomas [5]. This patient’s symptoms of daily headaches, nausea, and visual disturbances over two weeks were consistent with symptomatic pituitary apoplexy [1]. Empiric corticosteroid supplementation should be administered to all patients with signs of pituitary apoplexy [9,10]. Although the patient had a cortisol level of 12.5 mcg/dL, which is typically above the threshold for adrenal insufficiency, the profound hyponatremia and the presence of a macroadenoma with hemorrhage justified empiric glucocorticoid coverage [9-12]. Cosyntropin stimulation testing cannot be performed within three to six weeks of acute pituitary insults, as it assumes chronic adrenocorticotropic hormone (ACTH) deficiency with resultant adrenal atrophy, which is not the case in acute central adrenal insufficiency [13].

Surgical intervention is indicated if there is an optic nerve compromise [6]. Studies comparing surgical versus conservative management of pituitary apoplexy without optic nerve compromise show no significant difference in outcomes regarding visual acuity, visual field deficits, ocular paresis, or anterior pituitary hormonal abnormalities [14]. Following pituitary apoplexy, anterior pituitary hormone deficits are common, with partial or complete recovery reported in up to 50% of cases, but completely normal pituitary function in only 5-37% of patients [14]. Hyponatremia in this case may be related to glucocorticoid deficiency, which can cause SIADH and direct renal water excretion dysfunction. SIADH may also occur due to hypothalamic insult even in the absence of glucocorticoid deficiency [8,15]. Low serum bicarbonate levels in hyponatremia can help differentiate etiologies, with lower levels seen in adrenal insufficiency compared to SIADH [16].

The patient did not exhibit other pituitary deficits as she continued to lactate. Hyperprolactinemia was present but with levels less than 100 ng/mL, which is typical during breastfeeding or due to pituitary stalk deviation in macroadenomas [17]. Prolactin-secreting macroadenomas usually have levels greater than 200 ng/mL [18]. The patient's prolactin levels of 76-80 ng/mL may be artificially low due to the Hook effect; thus, a prolactin with dilution was recommended but not ordered by the care team due to unclear reasons [19]. While her thyroid function initially suggested central hypothyroidism with low thyroid stimulating hormone (TSH) and low total T3, her thyroid axis had normalized within a week. She did have very low estradiol, although she had recently received a medroxyprogesterone injection the previous month. 

## Conclusions

This case underscores the need for a high index of suspicion for pituitary pathology in women with severe hyponatremia and visual changes in the postpartum period. A multidisciplinary approach is essential for optimal management. Future research should focus on establishing clear guidelines for the management of pituitary apoplexy and associated endocrine abnormalities in postpartum. Early recognition and appropriate intervention are crucial to prevent long-term complications and ensure a favorable outcome.
